# Advocating for integrated preoperative nutritional and postoperative rehabilitative strategies in total knee arthroplasty

**DOI:** 10.1097/JS9.0000000000001784

**Published:** 2024-06-14

**Authors:** Rubing Lin, Zijian Li, Dan Shan

**Affiliations:** a Department of Orthopedics, Shenzhen Children’s Hospital, Shenzhen, Guangdong; b Medical School of Chinese PLA, Beijing, People’s Republic of China; c Clinical Science Institute, University Hospital Galway, Galway, Ireland

*Dear Editor*,

We read with interest the recent publication by Liu *et al*. in the *International Journal of Surgery* entitled ‘Associations of postoperative outcomes with geriatric nutritional risk index after conventional and robotic-assisted total knee arthroplasty: a randomized controlled trial’, which has provided significant insights into the interplay between robotic-assisted total knee arthroplasty (RA-TKA) and the nutritional status of elderly patients^1^. This study uniquely integrates the Geriatric Nutritional Risk Index (GNRI) with postoperative outcomes [e.g. Hip-Knee-Ankle (HKA) Angle Deviation, Knee Society Score (KSS), and Visual Analog Scale (VAS) for Pain], advancing our understanding of patient selection criteria for surgical techniques in orthopedics. However, while the study’s findings are groundbreaking, there are several aspects that warrant further discussion and exploration to refine the implications of GNRI in clinical settings.

Firstly, one of the most important observations by the authors is that the RA-TKA group does not significantly improve the postoperative KSS score in well-nourished patients (i.e. GNRI >100) as compared to the conventional TKA group, which raises questions about the cost-effectiveness of RA-TKA for the targeted individuals. This finding is particularly important as the cost and accessibility of robotic technology remain significant barriers to widespread adoption in clinical practice. Parallelly, two prior studies have also shown that while RA-TKA might enhance alignment accuracy, it may not always translate to improved functional outcomes compared to conventional methods^2,3^. Thus, the financial implications of choosing RA-TKA in settings where nutritional status is optimal should be thoroughly evaluated in future economic analyses. In addition, Liu *et al*. attribute the lack of significantly improved postoperative KSS in the RA-TKA group to the compensatory effect of good nutrition status among elderly adults with a GNRI greater than 100. However, other factors may also play a role. For instance, a well-structured postoperative rehabilitation program (e.g. physical therapy), particularly during the early recovery phase, could further significantly contribute to a similar outcome in KSS^4^. In other words, even if optimal alignment is achieved through RA-TKA, the absence of a suitable physical therapy and rehabilitation regimen could still limit a patient’s functional improvement.

Moreover, the study reports a substantial benefit of RA-TKA over conventional TKA in patients with relatively poor nutritional status (GNRI ≤100), indicated by improved KSS. This suggests that RA-TKA might be particularly advantageous for malnourished elderly populations, potentially mitigating the heightened risk of poor outcomes associated with their compromised health status. It is crucial, however, to investigate the underlying mechanisms through which RA-TKA confers greater benefits in this subgroup. Could improved precision in prosthesis placement reduce the systemic stress responses typically seen postoperatively in malnourished patients? Further mechanistic studies are needed to elucidate these pathways.

Additionally, the study underscores the necessity for comprehensive preoperative nutritional assessments to better categorize patients who are most likely to benefit from RA-TKA. This aligns with findings from previous studies^5,6^, which emphasized the predictive value of preoperative nutritional indices on surgical outcomes. Future research should focus on developing integrated care pathways that include nutritional optimization before RA-TKA to enhance postoperative recovery and long-term outcomes.

It may also be important to address the study’s limitations, particularly the small sample size of patients with GNRI ≤100 and the short-term follow-up period. The limited sample size may reduce the generalizability of the findings and potentially underpower the study to detect other clinically significant differences. Long-term follow-up studies would be beneficial to assess the durability of the prostheses and the long-term clinical outcomes, which are critical for substantiating the initial benefits observed in RA-TKA patients with low GNRI.

Furthermore, the authors’ focus on a Chinese population may limit the applicability of the results to other ethnicities and geographical locations. Orthopedic outcomes can vary significantly across different demographic groups due to variations in body habitus, lifestyle, and healthcare access^7–9^. Thus, expanding future studies to include diverse populations would enhance the external validity of the findings and help refine patient selection criteria for RA-TKA globally.

In conclusion, the study by Liu *et al*. is a commendable step forward in the quest to optimize surgical outcomes in knee arthroplasty through personalized patient care^1^. By incorporating GNRI, this research not only highlights the importance of nutritional assessment in elderly patients undergoing knee arthroplasty but also challenges us to rethink the value propositions of advanced surgical technologies like RA-TKA. We recommend a multidisciplinary approach involving orthopedic surgeons, geriatricians, and nutritionists to fully realize the benefits of this technology in clinical practice.

## Ethical approval

Not applicable.

## Consent

Not applicable.

## Source of funding

This study was supported by the Guangdong High-level Hospital Construction Fund and Sanming Project of Medicine in Shenzhen (SZSM202011012). The funders had no role in the design of the study, in the collection, analyses, or interpretation of data, in the writing of the manuscript, or in the decision to publish the results.

## Author contribution

R.L.: study design and writing the manuscript draft (leading); Z.L.: study design and writing the manuscript draft; D.S.: study design and manuscript revision.

## Conflicts of interest disclosure

The authors declare no conflicts of interest.

## Research registration unique identifying number (UIN)

Not applicable.

## Guarantor

Dan Shan.

## Data availability statement

Not applicable.

## Provenance and peer review

Not applicable.

## References

[1] Liu G Liu Q Tian R . Associations of postoperative outcomes with geriatric nutritional risk index after conventional and robotic-assisted total knee arthroplasty: a randomized controlled trial. Int J Surg (London, England) 2024;110:2115–2121. 10.1097/JS9.0000000000001048 PMC11019982 38241323

[2] Kim YH Yoon SH Park JW . Does robotic-assisted TKA result in better outcome scores or long-term survivorship than conventional TKA? A randomized, controlled trial. Clin Orthop Relat Res 2020;478:266–275. 31389889 10.1097/CORR.0000000000000916 PMC7438149

[3] Khan IA Vaile JR DeSimone CA . Image-free robotic-assisted total knee arthroplasty results in quicker recovery but equivalent one-year outcomes compared to conventional total knee arthroplasty. J Arthroplasty 2023;38(6S):S232–S237. 36801477 10.1016/j.arth.2023.02.023

[4] Konnyu KJ Thoma LM Cao W . Rehabilitation for total knee arthroplasty: a systematic review. Am J Phys Med Rehabil 2023;102:19–33. 35302953 10.1097/PHM.0000000000002008 PMC9464796

[5] Jiang X Xiang J Yang M . Predictive role of preoperative nutritional status on early postoperative outcomes in different-aged patients undergoing heart valve surgery. J Cardiothorac Vasc Anesth 2024;38:1169–1180. 38423886 10.1053/j.jvca.2024.01.037

[6] Huang W Xiao Y Wang H . Association of geriatric nutritional risk index with the risk of osteoporosis in the elderly population in the NHANES. Front Endocrinol 2022;13:965487. 10.3389/fendo.2022.965487 PMC9744963 36523597

[7] Elmallah RD Jauregui JJ Cherian JJ . Effect of age on postoperative outcomes following total knee arthroplasty. J Knee Surg 2016;29:673–678. 26838967 10.1055/s-0036-1571428

[8] Pandya NK Wustrack R Metz L . Current concepts in orthopaedic care sisparities. J Am Acad Orthop Surg 2018;26:823–832. 30180094 10.5435/JAAOS-D-17-00410

[9] Dy CJ Tipping AD Nickel KB . Variation in the delivery of inpatient orthopaedic care to Medicaid beneficiaries within a single Metropolitan Region. J Bone Joint Surg Am 2019;101:1451–1459. 31436652 10.2106/JBJS.18.01198 PMC7406144

